# A genetic association study reveals the relationship between the oral microbiome and anxiety and depression symptoms

**DOI:** 10.3389/fpsyt.2022.960756

**Published:** 2022-11-10

**Authors:** Chun'e Li, Yujing Chen, Yan Wen, Yumeng Jia, Shiqiang Cheng, Li Liu, Huijie Zhang, Chuyu Pan, Jingxi Zhang, Zhen Zhang, Xuena Yang, Peilin Meng, Yao Yao, Feng Zhang

**Affiliations:** Key Laboratory of Trace Elements and Endemic Diseases, School of Public Health, Collaborative Innovation Center of Endemic Disease and Health Promotion for Silk Road Region, Health Science Center, Xi'an Jiaotong University, Xi'an, China

**Keywords:** anxiety, depression, oral microbiome, Mendelian Randomization (MR), polygenic risk scores (PRS)

## Abstract

**Background:**

Growing evidence supports that alterations in the gut microbiota play an essential role in the etiology of anxiety, depression, and other psychiatric disorders. However, the potential effect of oral microbiota on mental health has received little attention.

**Methods:**

Using the latest genome-wide association study (GWAS) summary data of the oral microbiome, polygenic risk scores (PRSs) of 285 salivary microbiomes and 309 tongue dorsum microbiomes were conducted. Logistic and linear regression models were applied to evaluate the relationship between salivary-tongue dorsum microbiome interactions with anxiety and depression. Two-sample Mendelian randomization (MR) was utilized to compute the causal effects between the oral microbiome, anxiety, and depression.

**Results:**

We observed significant salivary-tongue dorsum microbiome interactions related to anxiety and depression traits. Significantly, one common interaction was observed to be associated with both anxiety score and depression score, *Centipeda periodontii SGB 224* × *Granulicatella uSGB 3289* (P _depressionscore_ = 1.41 × 10^−8^, P _anxietyscore_ = 5.10 × 10^−8^). Furthermore, we detected causal effects between the oral microbiome and anxiety and depression. Importantly, we identified one salivary microbiome associated with both anxiety and depression in both the UKB database and the Finngen public database, *Eggerthia* (P _IVW − majordepression − UKB_ = 2.99 × 10^−6^, P _IVW − Self − reportedanxiety/panicattacks − UKB_ = 3.06 × 10^−59^, P _IVW − depression − Finngen_ = 3.16 × 10${}_{,}^{-16}$ P _IVW − anxiety − Finngen_ = 1.14 × 10^−115^).

**Conclusion:**

This study systematically explored the relationship between the oral microbiome and anxiety and depression, which could help improve our understanding of disease pathogenesis and propose new diagnostic targets and early intervention strategies.

## Introduction

Anxiety disorders and depression are prevalent mental illnesses, with an estimated 264 million people suffering from anxiety disorders and 322 million people suffering from depression in 2015, according to the World Health Organization (WHO) global health estimates (1). As represented in the Global Burden of Disease Study 2013 (GBD 2013), the second most common cause of years lived with disability (YLDs) was depression, and the ninth was anxiety (2). Furthermore, according to the description of the GBD Compare, the disability-adjusted life years (DALYs) for depression were 1.84 and 1.13 for anxiety (https://vizhub.healthdata.org/gbd-compare/). In conclusion, the high prevalence of anxiety disorders and depression and the huge burden and harm caused by both diseases to society and individuals have made anxiety and depression highly concerning. Previous studies have found that human genetics regulates the pathogenesis of anxiety and depression (3, 4). Offspring of patients suffering from anxiety were 4–6 times more likely to develop anxiety than offspring of the general population (3). In twin studies, the heritability of anxiety disorders was approximately 30–50% (3), and the heritability of depression was approximately 35% (4).

The microbiota is a complex ecosystem of microorganisms containing viruses, fungi, bacteria, and protozoa that live in different parts of the human body, such as the mouth, the gastro-enteric tube, the vagina, the respiratory system, and the skin (5). The microbiota plays an essential role in the host immune system's induction, cultivation, and function. Accordingly, the host immune system has developed multiple ways to maintain its symbiotic relationship with the microbiota (6). More than 70% of microbiota are found in the gastrointestinal tract and maintain a mutually beneficial relationship with the host (6). As a complex physiological ecosystem, the gut microbiota influences its host health. Growing evidence links the gut microbiota to various psychiatric and neurological disorders, such as schizophrenia, depression, bipolar disorder, autism spectrum disorder, Alzheimer's disease, and Parkinson's disease (7–12). Studies have shown that the links between the gut microbiota and these diseases may be due to a bidirectional communication system between the gut and the central nervous system (CNS) called the “microbiota-gut-brain axis” (MGBA) (13, 14). The signaling mechanism of the bidirectional communication system is that the brain regulates gut function *via* the hypothalamic-pituitary-adrenal (HPA) axis and the autonomic nervous system. For example, the brain releases norepinephrine during stress, and norepinephrine has been found to stimulate the proliferation of gut pathogens (15, 16).

Furthermore, the gut modulates CNS function through a variety of microbiota-derived metabolites and products. For instance, gut hormones and neuroactive substances are delivered to the brain *via* the circulatory system, enteric nervous system, immune system, and vagus nerve (14). In recent years, the diversity of microbiota species in the mouth has made it the focus of research. Several mechanisms may exist for the link between poor oral health and mental health disorders, such as changes in the oral microbiota (17). Nevertheless, the relationship between the oral microbiome and anxiety and depression remains unclear.

Previously, researchers have revealed the impact of human genetics on the oral microbiome (18). A twin oral microbiome study showed that the oral microbiome was heritable, and the heritability of many microbiome phenotypes was more than 50% (19). Although the effect of host genetics on the composition and stability of the oral microbiome remains poorly understood, previous GWAS studies have identified several genetic loci associated with the oral microbiome. For example, an unbiased GWAS analysis by Demmitt et al. (19) suggested that the genes IMMPL2 and INHBA-AS1 could influence the oral microbiome. The study by Poole et al. (20) identified AMY1-CN as a genetic factor associated with microbial composition and function. However, these findings are still limited. Polygenic risk scores (PRSs) provide an overall estimate of the genetic predisposition for a trait at the individual level by calculating the sum of the effects of risk alleles, where each risk allele estimates the phenotype from an independent GWAS (21). Previously, PRS has also been used to assess the impact of d dietary habits and the gut microbiome on anxiety and depression (22).

This study conducted polygenic risk scores (PRSs) analysis of the salivary microbiome and tongue dorsum microbiome in the UK Biobank cohort. Logistic/linear regressions were applied to analyze the associations between salivary microbiome-PRSs, tongue dorsum microbiome-PRSs, and their interactions with anxiety and depression. Then, Mendelian Randomization (MR) was used to evaluate the causal relationship between each salivary microbiome, tongue dorsum microbiome, anxiety, and depression.

## Materials and methods

### The UK Biobank cohort and the definition of anxiety and depression

The UK Biobank (UKB) (http://www.ukbiobank.ac.uk) recruited 502,656 participants aged 40–69 from 2006 to 2010 and recorded health information, hospital records, and genetic data of participants (23). UKB performed genotyping, imputation, and quality control (QC) for 487,409 individuals. For example, the UK Bileve axiom array and the UK Biobank axiom array were used for genotyping. Both arrays share over 95% of their marker content. IMPUTE4 was used for imputation in chunks of about 50,000 imputed markers with a 250 kb buffer region. QC, imputation, and post-imputation cleaning were performed centrally by UKB. Participants were excluded who were inconsistent between self-reported gender and genetic gender, without imputation data and ethical consent. Additionally, all genetically related individuals were removed by the KING software (http://people.virginia.edu/~wc9c/KING/) (24). More information about genotyping, imputation, QC, and physical measurements has been described previously (23). Our research has been approved by the UK Biobank (Application 46478). The UK Biobank has support from the North West Multi-center Research Ethics Committee (MREC) and the Human Tissue Authority (HTA). All participants agreed to use their anonymous data and samples for any health-related studies and to reconnect for further sub-studies (25).

The study obtained two common psychiatric disorders, including anxiety (self-reported anxiety and general anxiety disorder (GAD-7) scores) and depression (self-reported depression and the patient health questionnaire (PHQ-9) scores). Both anxiety and depression are derived from UKB. For anxiety, the present study included 155 076 participants with GAD-7 scores and 138,709 participants with self-reported anxiety status data (27,898 cases and 110,811 controls). For depression, 154,360 participants with PHQ-9 scores and 157,459 participants with self-reported depression status data (76,672 cases and 80,787 controls) were included in this study. The basic characteristics of the study subjects and detailed information are presented in Table 1.

**Table 1:** The basic characteristics of study samples.

	**Depression**		**Anxiety**	
	**PHQ-9**	**Self-reported depression**	**GAD-7**	**Self-reported anxiety**
Participants	154 360	157 459 (76 672 cases/ 80 787 controls)	155 076	138 709 (27 898 cases/ 110 811 controls)
Females, n (%)	87 206 (56.50)	89 746 (57.00)	87 604 (56.49)	77,088 (55.58)
Mean age (SD)	55.90 (7.74)	56.11 (7.78)	55.89 (7.74)	56.16 (7.70)

PHQ, Patient Health Questionnaire; GAD, Generalized Anxiety Disorder.

### GWAS summary datasets of the oral microbiome

The GWASs of 309 tongue dorsum microbiomes (*N* = 2,017) and 285 salivary microbiomes (*N* = 1,915) were obtained from a recently published metagenome-genome-wide association study of the human oral microbiome (18). The conservative inclusion threshold of mean depth > 8× , Hardy Weinberg equilibrium (HWE) > 10^−5^, and genotype calling rate > 98% for variants were applied. Furthermore, the samples had to meet the following criteria: variant calling rate > 98%, mean sequencing depth > 20× , no population stratification in principal component analysis (PCA), and excluding related individuals by calculating pairwise identity by descent. After quality control, 2,984 individuals (2,017 tongue dorsum and 1,915 salivary) with about 10 million common and low-frequency (MAF ≥ 0.5%) variants were contained. More detailed information on sample collection, sequencing, microbiome trait preparation, observational analysis, and genotyping analysis is described elsewhere (18).

### Oral microbiome-related PRSs calculation

We calculated the oral microbiome-related PRSs of each subject based on the individual genotype data of UKB by using PLINK2.0. For oral microbiome PRS, PRS_n_ denotes the PRS value of the oral microbiome for the nth subject, defined as follows:


$$PRS_{n}=\sum_{i=1}^{l}E_{i}D_{in},$$

where l denotes the total number of gut microbiota-associated SNPs; E_i_ denotes the effect size of significant gut microbiota-associated SNP i; and D_in_ denotes the dosage of the risk allele of the ith SNP for the nth individual (0 is coded for homozygous protective genotype, one for heterozygous, and two for homozygous polymorphic genotypes).

### Statistical analysis

The logistic and linear regression models were used to evaluate the associations of tongue dorsum microbiome-PRSs, salivary microbiome-PRSs, and their interactions with depression and anxiety. The logistic and linear regression models were established by R software (R-4.1.2). The tongue dorsum microbiome-PRSs, salivary microbiome-PRSs, and salivary microbiome PRSs × tongue dorsum microbiome-PRSs were selected as independent variables; PHQ-9 score, self-reported depression, GAD-7 score, and self-reported anxiety were fitted as dependent variables; sex, age, 10PC, ever smoked, ever drank, and Townsend deprivation index (TDI) were set as covariates. Bonferroni corrected the *P* value of 1.42×10^−7^ [*P* = 0.05/ (285×309×4)] as the significance threshold for correcting the multiple testing.

Two-sample MR analysis was used to assess the causal relationship between the tongue dorsum microbiome, salivary microbiome, anxiety, and depression. The tongue dorsum and salivary microbiome were selected as instrumental variables (IVs), and anxiety and depression were used as the outcome variables. The inverse variance weighted (IVW) was used as the primary causal effect estimate. The IVW method is an ideal estimation, and it is an effective analysis under the assumption that all genetic variants are effective instrumental variables, and it has a strong ability to detect causality (26). But the IVW specifically requires that genetic variants affect the target outcome only through exposure in the study. Although known confounding SNPs were excluded as much as possible in this study, there were still many unknown confounders that could lead to pleiotropy and biased effect size estimates. Therefore, we adopted two methods to check the reliability and stability of the results, namely the MR Egger regression (27) and the Weighted Median Estimator (WME) (28). Bonferroni corrected the *P*-value of 2.84× 10^−7^ [*P* = 0.05/ (285×309×2)] as the significance threshold for correcting the multiple testing. The MR analysis was performed using the “Two-Sample MR” package for R 3.5.3 (29).

This study used the IVW and MR Egger regressions to test the heterogeneity. If the *P* > 0.05, it was considered that there was no heterogeneity in the included IVs, and the effect of heterogeneity on the estimation of causal effects could be ignored. Furthermore, MR Egger regression was used to evaluate the bias caused by horizontal pleiotropy, and the Egger intercept can evaluate the size of pleiotropy. In this study, the P-value of the pleiotropy test was used to measure whether there was pleiotropy in the analysis. If *P* > 0.05, the possibility of pleiotropy in the causal effect is considered to be weak, and its impact can be ignored. Furthermore, GWAS summary statistics of anxiety and depression in the Finngen public database (https://www.finngen.fi/en/access_results) were used to verify the causal effects between tongue dorsum microbiome, salivary microbiome, anxiety, and depression in UKB.

## Results

### Associations of the oral microbiome with depression

**Figure 1:**
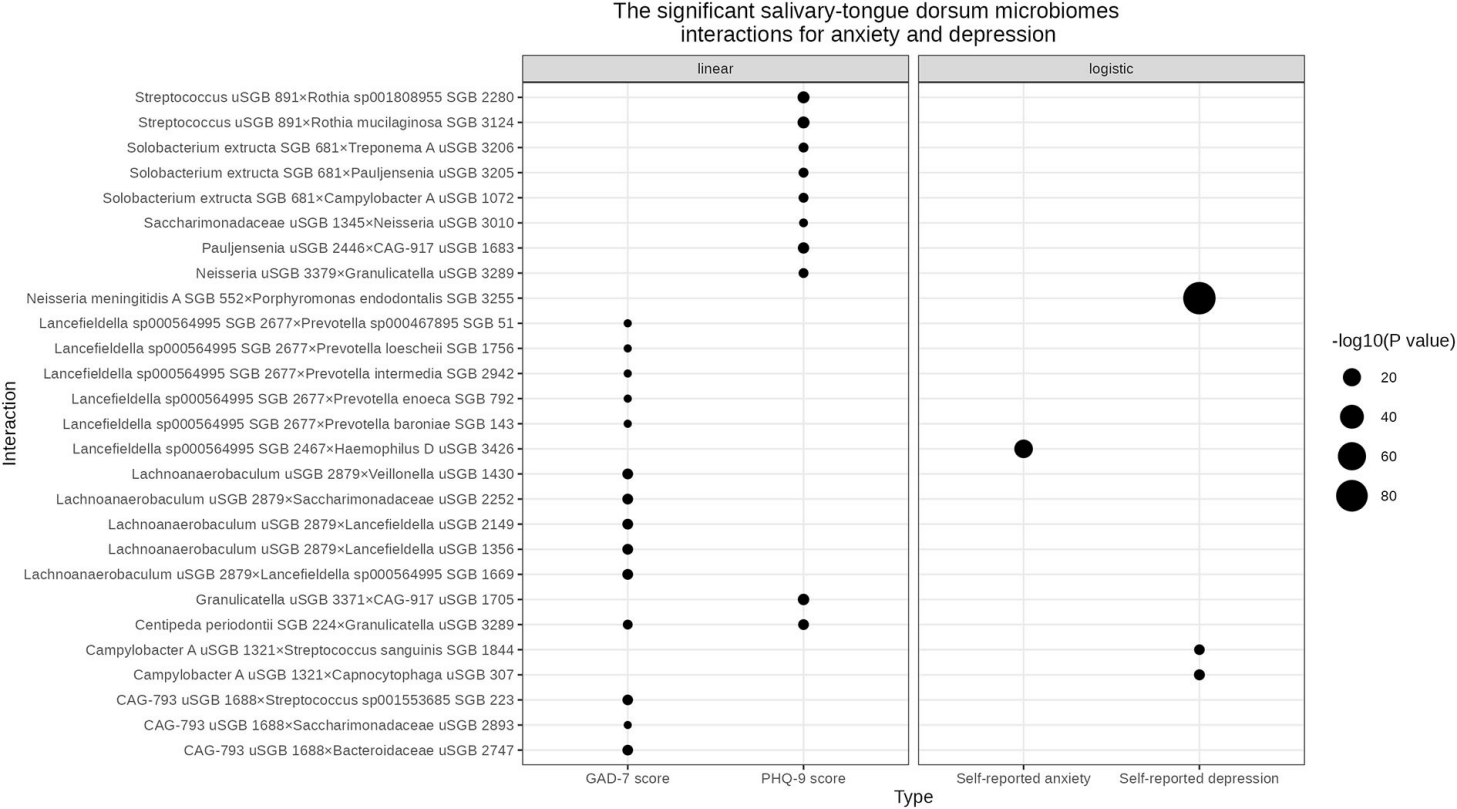
The significant salivary-tongue dorsum microbiome interactions for anxiety and depression. The bubble chart represents the significant salivary-tongue dorsum microbiome interactions for anxiety and depression in the linear regression model and logistic regression model, respectively. Circle size indicates the -log10 (P value) of each interaction.

We found three and 10 significant salivary-tongue dorsum microbiome interactions for self-reported depression and PHQ-9 score, respectively (Table 2). Such as *Streptococcus unclassified SGB (uSGB) 891* × *Rothia mucilaginosa SGB 3124* (P _PHQ − 9score_ = 1.34 × 10^−9^) and *Campylobacter A uSGB 1321* × *Capnocytophaga uSGB 307* (P _self − reporteddepression_ = 1.03 × 10^−8^). After integrating the two results, no common interactions were shared between the PHQ-9 score and self-reported depression. After gender stratification, we identified five significant interactions for the PHQ-9 score in females and 12 significant interactions for the PHQ-9 score in males (Supplementary Tables S1, S2), such as *Gemella morbillorum SGB 349* × *Solobacterium uSGB 2587* (P _PHQ − 9−female_ = 9.81 × 10^−10^). The significant salivary-tongue dorsum microbiome interactions for depression are shown in Figure 1.

**Table 2:** The significant salivary-tongue dorsum microbiomes interactions for self-reported depression and PHQ-9 score.

**Interaction (salivary microbiomes × tongue dorsum microbiomes)**	**Beta**	**SE**	***P*** **value**
**Self-reported depression**			
Campylobacter A uSGB 1321 × Capnocytophaga uSGB 307	6.50 × 10^14^	1.13 × 10^14^	1.03 × 10^−8^
Campylobacter A uSGB 1321 × Streptococcus sanguinis SGB 1844	1.04 × 10^15^	1.85 × 10^14^	1.79 × 10^−8^
Neisseria meningitidis A SGB 552 × Porphyromonas endodontalis SGB 3255	1.95 × 10^12^	9.95 × 10^10^	1.05 × 10^−85^
**PHQ-9 score**			
Streptococcus uSGB 891 × Rothia mucilaginosa SGB 3124	0.064	0.011	1.34 × 10^−9^
Streptococcus uSGB 891 × Rothia sp001808955 SGB 2280	0.064	0.011	1.34 × 10^−9^
Granulicatella uSGB 3371 × CAG-917 uSGB 1705	0.042	0.007	4.21 × 10^−9^
Pauljensenia uSGB 2446 × f CAG-917 uSGB 1683	−0.021	0.004	5.24 × 10^−9^
Centipeda periodontii SGB 224 × Granulicatella uSGB 3289	0.021	0.004	1.41 × 10^−8^
Solobacterium extructa SGB 681 × Treponema A uSGB 3206	−963.461	175.805	4.25 × 10^−8^
Solobacterium extructa SGB 681 × Pauljensenia uSGB 3205	302.352	55.209	4.35 × 10^−8^
Solobacterium extructa SGB 681 × Campylobacter A uSGB 1072	−596.774	109.062	4.46 × 10^−8^
Neisseria uSGB 3379 × Granulicatella uSGB 3289	8.727	1.597	4.62 × 10^−8^
Saccharimonadaceae uSGB 1345 × Neisseria uSGB 3010	−8.72 × 10^9^	1.64 × 10^9^	1.05 × 10^−7^

PHQ, Patient Health Questionnaire.

### Associations of the oral microbiome with anxiety

One significant salivary-tongue dorsum microbiome interaction was associated with self-reported anxiety, and 14 interactions were related to the GAD-7 score (Table 3). Such as *Lancefieldella sp000564995 SGB 2467*× *Haemophilus D uSGB 3426* (P _Self − reportedanxiety_ = 5.50× 10^−22^) and *Lachnoanaerobaculum uSGB 2879*× *Lancefieldella uSGB 1356* (P _GAD − 7score_ = 1.42× 10^−8^). No common interaction was shared by both the GAD-7 score and self-reported anxiety. After gender stratification, 105 significant interactions were found for the GAD-7 score in males (Supplementary Table S3). The significant salivary-tongue dorsum microbiome interactions for anxiety are shown in Figure 1.

**Table 3:** The significant salivary-tongue dorsum microbiomes interactions for self-reported anxiety and GAD-7 score.

**Interaction (salivary microbiomes × tongue dorsum microbiomes)**	**Beta**	**SE**	***P*** **value**
**Self-reported anxiety**			
Lancefieldella sp000564995 SGB 2467 × Haemophilus D uSGB 3426	−6.83 × 10^15^	7.08 × 10^14^	5.50 × 10^−22^
**GAD-7 score**			
Lachnoanaerobaculum uSGB 2879 × Lancefieldella uSGB 1356	−0.074	0.013	1.42 × 10^−8^
Lachnoanaerobaculum uSGB 2879 × Veillonella uSGB 1430	−0.074	0.013	1.42 × 10^−8^
Lachnoanaerobaculum uSGB 2879 × Lancefieldella sp000564995 SGB 1669	−0.074	0.013	1.42 × 10^−8^
Lachnoanaerobaculum uSGB 2879 × Saccharimonadaceae uSGB 2252	−0.074	0.013	1.42 × 10^−8^
Lachnoanaerobaculum uSGB 2879 × Lancefieldella uSGB 2149	−0.074	0.013	1.42 × 10^−8^
CAG-793 uSGB 1688 × Bacteroidaceae uSGB 2747	−166.356	29.462	1.64 × 10^−8^
CAG-793 uSGB 1688 × Streptococcus sp001553685 SGB 223	486.829	86.559	1.87 × 10^−8^
Centipeda periodontii SGB 224 × Granulicatella uSGB 3289	0.019	0.003	5.10 × 10^−8^
Lancefieldella sp000564995 SGB 2677 × Prevotella baroniae SGB 143	0.077	0.015	1.25 × 10^−7^
Lancefieldella sp000564995 SGB 2677 × Prevotella enoeca SGB 792	0.077	0.015	1.25 × 10^−7^
Lancefieldella sp000564995 SGB 2677 × Prevotella intermedia SGB 2942	0.077	0.015	1.25 × 10^−7^
Lancefieldella sp000564995 SGB 2677 × Prevotella loescheii SGB 1756	0.077	0.015	1.25 × 10^−7^
Lancefieldella sp000564995 SGB 2677 × Prevotella sp000467895 SGB 51	0.077	0.015	1.25 × 10^−7^
CAG-793 uSGB 1688 × Saccharimonadaceae uSGB 2893	−222.061	42.009	1.25 × 10^−7^

GAD is a general anxiety disorder.

### The common oral microbiome is associated with depression and anxiety

We discovered one significant common salivary-tongue dorsum microbiome interaction shared by the PHQ-9 score and GAD-7 score, *Centipeda periodontii SGB 224* × *Granulicatella uSGB 3289* (P _PHQ − 9_ = 1.41 × 10^−8^, P _GAD − 7_ = 5.10 × 10^−8^) (Supplementary Table S4). After gender stratification, no significant common interactions were shared between anxiety and depression.

### The causal relationship between the oral microbiome and depression

**Figure 2:**
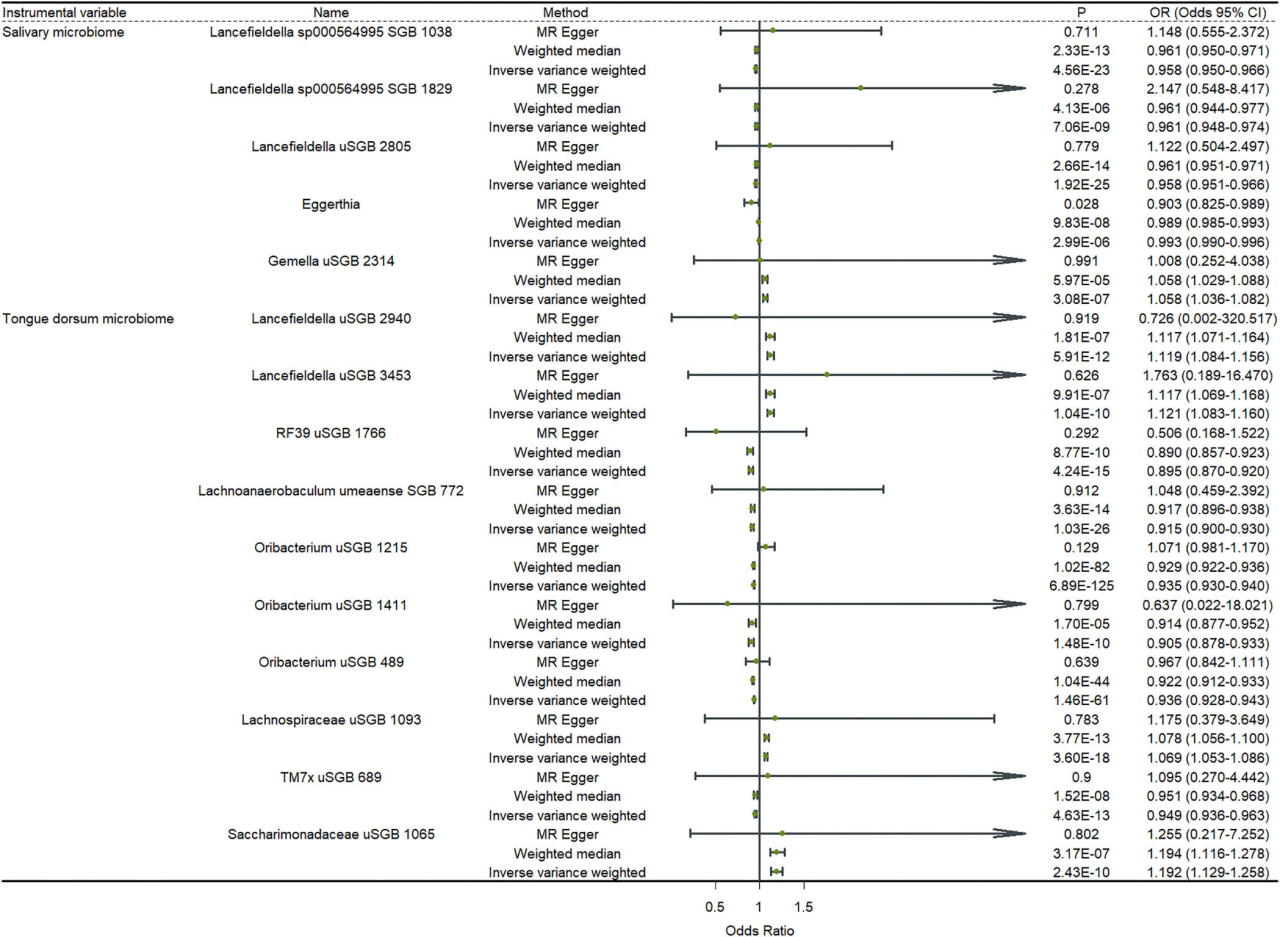
Causal effects of the oral microbiome on depression. Results from inverse-variance weighted (IVW), MR-Egger, and weighted median. Error bars indicate 95% confidence intervals.

Figure 2 shows the results of a two-sample MR analysis identifying the relationship between salivary microbiomes, tongue microbiomes, and major depression. We observed that five salivary microbiomes and 10 tongue dorsum microbiomes were the causal risk factors for major depression in the UK Biobank. Furthermore, we discovered that four salivary microbiomes and six tongue dorsum microbiomes were causal risk factors for depression in the Finngen public database (Supplementary Table S5). Both the UK Biobank and Finngen public databases shared one common salivary microbiome and five tongue dorsum microbiomes as the risk factors for depression. Such as *Eggerthia* in salivary microbiomes (P _UKB − IVW_ = 2.99 × 10^−6^, P _UKB − WME_ = 9.83 × 10^−8^, P _Finngen − IVW_ = 3.16 × 10^−16^, and P _Finngen − WME_ = 1.43 × 10^−16^) and *Lancefieldella uSGB 2940* in tongue dorsum microbiomes (P _UKB − IVW_ = 5.91 × 10^−12^, P _UKB − WME_ = 1.81 × 10^−07^, P _Finngen − IVW_ = 1.06 × 10^−18^, and P _Finngen − WME_ = 1.48 × 10^−10^). The estimated cause-effect sizes of the SNPs on both the exposure (salivary microbiomes and tongue dorsal microbiomes) and outcome (depression) were displayed in scatter plots (Supplementary Figures S1, S2).

### The causal relationship between the oral microbiome and anxiety

**Figure 3:**
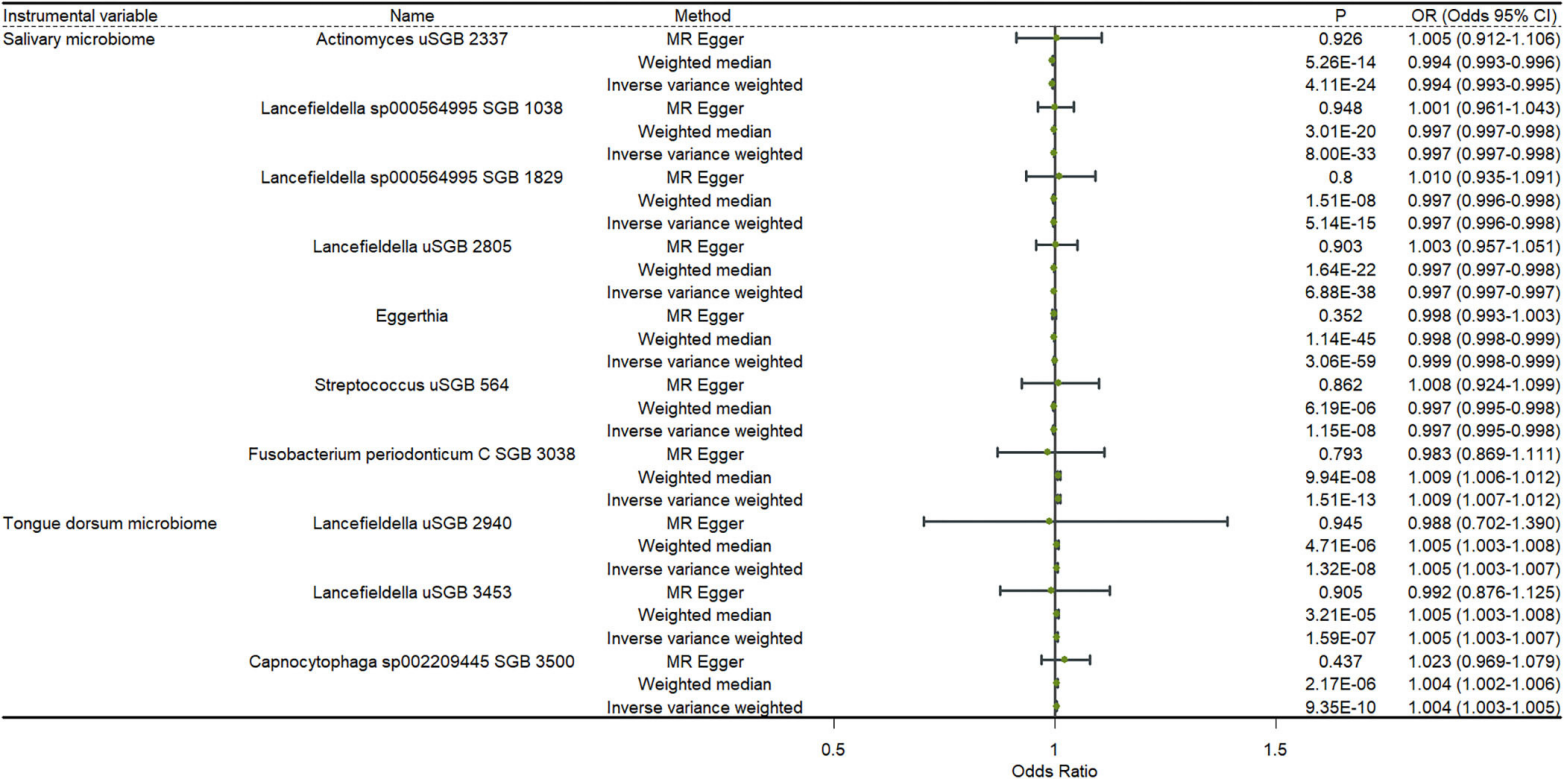
Causal effects of the oral microbiome on anxiety. Results from inverse-variance weighted (IVW), MR-Egger, and weighted median. Error bars indicate 95% confidence intervals.

Figure 3 shows the results of a two-sample MR analysis identifying the relationship between salivary microbiomes, tongue dorsum microbiomes, and anxiety. We observed that seven salivary microbiomes and three tongue dorsum microbiomes were the causal risk factors for anxiety in UK Biobank. Furthermore, we discovered that six salivary microbiomes and four tongue dorsum microbiomes were causal risk factors for anxiety in the Finngen public database (Supplementary Table S6). Both the UK Biobank and Finngen public databases shared four common salivary microbiomes and one tongue dorsum microbiome as the risk factors for anxiety. Such as *Actinomyces uSGB 2337* in salivary microbiomes (P _UKB − IVW_ = 4.11 × 10^−24^, P _UKB − WME_ = 5.26 × 10^−14^, P _Finngen − IVW_ = 4.10 × 10^−30^, P _Finngen − WME_ = 1.82 × 10^−18^) and *Capnocytophaga sp002209445 SGB 3500* in tongue dorsum microbiomes (P _UKB − IVW_ = 9.35 × 10^−10^, P _UKB − WME_ = 2.17 × 10^−06^, P _Finngen − IVW_ = 2.25 × 10^−8^, and P _Finngen − WME_ = 6.26 × 10^−6^). The estimated cause-effect sizes of the SNPs on both the exposure (salivary microbiomes and tongue dorsal microbiomes) and outcome (anxiety) were displayed in scatter plots (Supplementary Figures S3, S4).

### The common causal relationship between anxiety and depression

Supplementary Table S7 displayed six shared risk factors between major depression and self-reported anxiety/panic attacks of UKB, including four salivary microbiomes and two dorsal tongue microbiomes. Supplementary Table S8 showed five shared risk factors between depression and anxiety in the Finngen database, including three salivary microbiomes and two dorsal tongue microbiomes. Concurrently, we detected a salivary microbiome to be a risk factor for all anxiety, and depression in the UKB and Finngen databases, *Eggerthia* (P _IVW − majordepression − UKB_ = 2.99 × 10^−6^, P _IVW − Self − reportedanxiety/panicattacks − UKB_ = 3.06 × 10^−59^, P _IVW − depression − Finngen_ = 3.16 × 10${}_{,}^{-16}$ and P _IVW − anxiety − Finngen_ = 1.14 × 10^−115^).

## Discussion

In the present study, we used logistic and linear regression models to find that multiple significant saliva-dorsal tongue microbiome interactions were associated with depression/anxiety. Furthermore, we found causal associations between the salivary microbiomes, dorsal tongue microbiomes, anxiety, and depression with the MR analysis, and these associations were confirmed in the Finngen public database.

It is no secret that dysbiosis of the oral microbiome can cause oral diseases like decayed teeth and periodontitis (30). However, a recent study showed that adolescent anxiety and depression symptoms were associated with the differential abundance of specific oral bacterial taxa, including *Actinomyces, Spirochaetaceae, Fusobacterium*, and *Treponema* (31). Moreover, Wingfield et al. (32) examined the structure and composition of the salivary microbiome in young adults with depression and a control group. They revealed that 21 bacterial taxa differed in abundance in the depressed cohort, including increased *Neisseria spp*. and *Prevotella nigrescens*, while the abundance of 19 taxa decreased. Although both studies have confirmed the relationship between the oral microbiome and depression, limited research has explained the specific mechanism. A previous study (33) has shown that periodontal bacteria can directly arrive in the brain through the bloodstream or areas with an incomplete or damaged blood-brain barrier (BBB). In addition, periodontitis can indirectly affect the central nervous system through pro-inflammatory cytokines (33). Pro-inflammatory cytokines activate endothelial cells to express TNF-α and interleukin-1 (IL-1) receptors, transmitting signals to perivascular macrophages that activate microglia, leading to neuroinflammation. Moreover, periodontitis can also lead to leaky periodontium and lipopolysaccharides (LPS) in the systemic circulation, activating the HPA axis, and increasing stress hormones or neurotransmitters (33).

In the dorsal tongue microbiome, we found an interesting bacterial taxon, *Porphyromonas*. *Porphyromonas gingivalis* (Pg) is a gram-negative anaerobic pathogen of periodontitis that produces LPS, gingipain, and capsules that damage local periodontal tissue (34). In the etiology of periodontitis, Pg was considered to be the major pathogen. Numerous cross-sectional epidemiological studies have shown that the incidence of periodontitis is positively associated with the incidence of depression (35, 36). However, their causality and latent mechanisms are largely unknown. Nowadays, the neurotrophin deficiency hypothesis of depression has received extensive attention (37). The hypothesis revealed that reducing neurotrophic factors makes the brain unable to adapt to environmental stimulation, which contributes to the onset of depression (37). Brain-derived neurotrophic factor (BDNF) is an important member of the neurotrophic factor family and plays a key role in the formation and plasticity of neuronal networks (37). According to research, infusion of BDNF into the midbrain or the dentate gyrus (DG) of the hippocampus increases antidepressant-like behavior in mice (38). A recent study has shown that Pg is an underlying risk factor for depression. They injected female mice with Pg every other day for 4 weeks and found that the Pg mice exhibited marked depression-like behavior (39). The possible mechanism is to increase activation of astrocytes in the hippocampus through the Pg-LPS/TLR4 signaling pathway, leading to downregulating astrocyte p75NTR and inhibiting BDNF maturation and, ultimately, depression. In addition, another study (40) also shown that Pg-LPS induces cognitive dysfunction mediated by neuronal inflammation *via* activation of the TLR4 signaling pathway in C57BL/6 mice.

Moreover, we also found some bacterial taxa, such as *Actinobacteria* and *Firmicutes*, live both in the oral cavity and the gut. Surprisingly, whether in the oral cavity or the gut, those bacterial taxa have been linked to depression (31, 41, 42). This may be because, although the composition of the microbiome is site-specific, there was evidence of some degree of overlap and crosstalk between the oral and gut microbiomes, and the oral bacteria may colonize the gut and cause chronic inflammation (43). In addition, microbes and their metabolites in the oral cavity were also likely to migrate or leak into the compromised BBB, leading to neuroinflammation, an important feature of the etiology of depression (44). In conclusion, it is known that periodontal pathogens are closely related to the etiology and pathophysiology of neuropsychiatric disorders such as depression and schizophrenia, especially immune system dysregulation, which plays an integral role in the etiology and pathophysiology of these diseases (33). Therefore, we can focus on the oral microbiota associated with periodontal disease as a target for future therapeutic interventions to alleviate the symptoms of these debilitating psychiatric disorders. In addition, maintaining a healthy oral microbiome is expected to help improve general and mental health, so we can take some probiotics to prevent mental illness.

At the family level, *Actinomycetaceae* have also been linked to anxiety and depression (31). Existing research hypothesizes that salivary cortisol is elevated in mental health disorders due to dysregulated HPA axis activity, which may be associated with psychological symptoms and oral bacterial abundance (45). Simpson et al. (31) reported that cortisol regulated the relationship between anxiety and depression symptoms with a variety of microbial taxa. For example, they found that participants with high anxiety symptoms and above-average basal cortisol levels had significantly lower *Actinomyces*, but the relationship was not observed in participants with high anxiety levels but below average cortisol levels. Moreover, significant interactions were also observed at the order and family levels (*Actinomycetaceae* and *Actinomycetales*). Furthermore, at the Phylum level, *Firmicutes* were consistently proven to be associated with Alzheimer's disease (AD) (46).

After gender stratification, we found significant differences in the salivary-tongue dorsum microbiome interaction between males and females. For the PHQ-9 score, we found five interactions in females and 12 in males; for the GAD-7 score, we found 105 significant interactions in males. This suggested that the effects of the oral microbiome on anxiety and depression were different in males and females. This may be because of gender differences in the oral microbiome. Previous evidence suggested that oral and gut microbiota composition can be regulated by estrogen levels (47), and estrogen receptor-β has been identified in the oral mucosa and salivary glands (48). A previous study (49) analyzed the oral microbiota composition in fasted and fed states in 20 subjects (10 women/10 men). In addition, increased relative abundances of the family *Pasteurellacae* and the genus *Haemophilus* in the women were observed in the fed condition. The genus *Capnocytophaga* was significantly more abundant in the male subjects. In the fasted condition, the main difference between men and women was for the genus *Eikenella*, which is more abundant in male subjects. Their study showed that men have distinct oral microbiota compared to women in fed or fasted conditions, which may be related to the glycemic response after feeding.

There were several strengths and limitations in the present study. First, this study was based on a large number of study samples, reducing errors due to the small sample size. Second, we utilized the latest GWAS data on the oral microbiome (saliva microbiome and dorsal tongue microbiome), as well as the UK Biobank Cohort genotype data, the results of which improve the ability to detect significant interactions. Finally, we also used data from the Finngen database to verify the causal relationship between oral microbiota and anxiety and depression in the UK Biobank. However, this study still has several limitations that cannot be ignored. First, our results could have been affected by a possible confounding bias because of the impact of various factors on mental disorders, such as early adversity and comorbid illness. Second, changes in host behavior, such as nutrition, can alter the oral microbiome, and mental health conditions like anxiety and depression have also been linked to dietary changes. So, changes in anxiety, depression, and oral microbiome are complex and require further research. Most important, the GWASs data for the oral microbiota in this study were derived from Asian populations, while the genotype data and the GWASs data of anxiety and depression of UK Biobank were from European ancestry, so our findings may not be generalizable across ethnic groups.

In conclusion, despite these limitations, this study systematically explored the relationship between oral microbiota and anxiety and depression. This work highlights the need for more research on the potential role of the oral microbiome in mental health disorders to improve our understanding of disease pathogenesis, potentially leading to new diagnostic targets and early intervention strategies.

## Data availability statement

The datasets analyzed during the current study are available in the UK Biobank [https://www.ukbiobank.ac.uk] and the CNGB [https://db.cngb.org/search/project/CNP0001664].

## Ethics statement

Ethical review and approval was not required for the study on human participants in accordance with the local legislation and institutional requirements.

## Author contributions

All authors listed have made a substantial, direct, and intellectual contribution to the work and approved it for publication.

## Funding

This work was supported by the National Natural Scientific Foundation of China [81922059] and the Natural Science Basic Research Plan in Shaanxi Province of China [2021JCW-08].

## Conflict of interest

The authors declare that the research was conducted in the absence of any commercial or financial relationships that could be construed as a potential conflict of interest.

## Publisher's note

All claims expressed in this article are solely those of the authors and do not necessarily represent those of their affiliated organizations, or those of the publisher, the editors and the reviewers. Any product that may be evaluated in this article, or claim that may be made by its manufacturer, is not guaranteed or endorsed by the publisher.
