# Supplementary material for: A genetic association study reveals the relationship between the oral microbiome and anxiety and depression symptoms
Source: Front Psychiatry. 2022 Nov 10;13:960756. doi: 10.3389/fpsyt.2022.960756 (PMC9685528; doi:10.3389/fpsyt.2022.960756)
Supplement: Supplementary file 1 [file Table_1.DOCX]

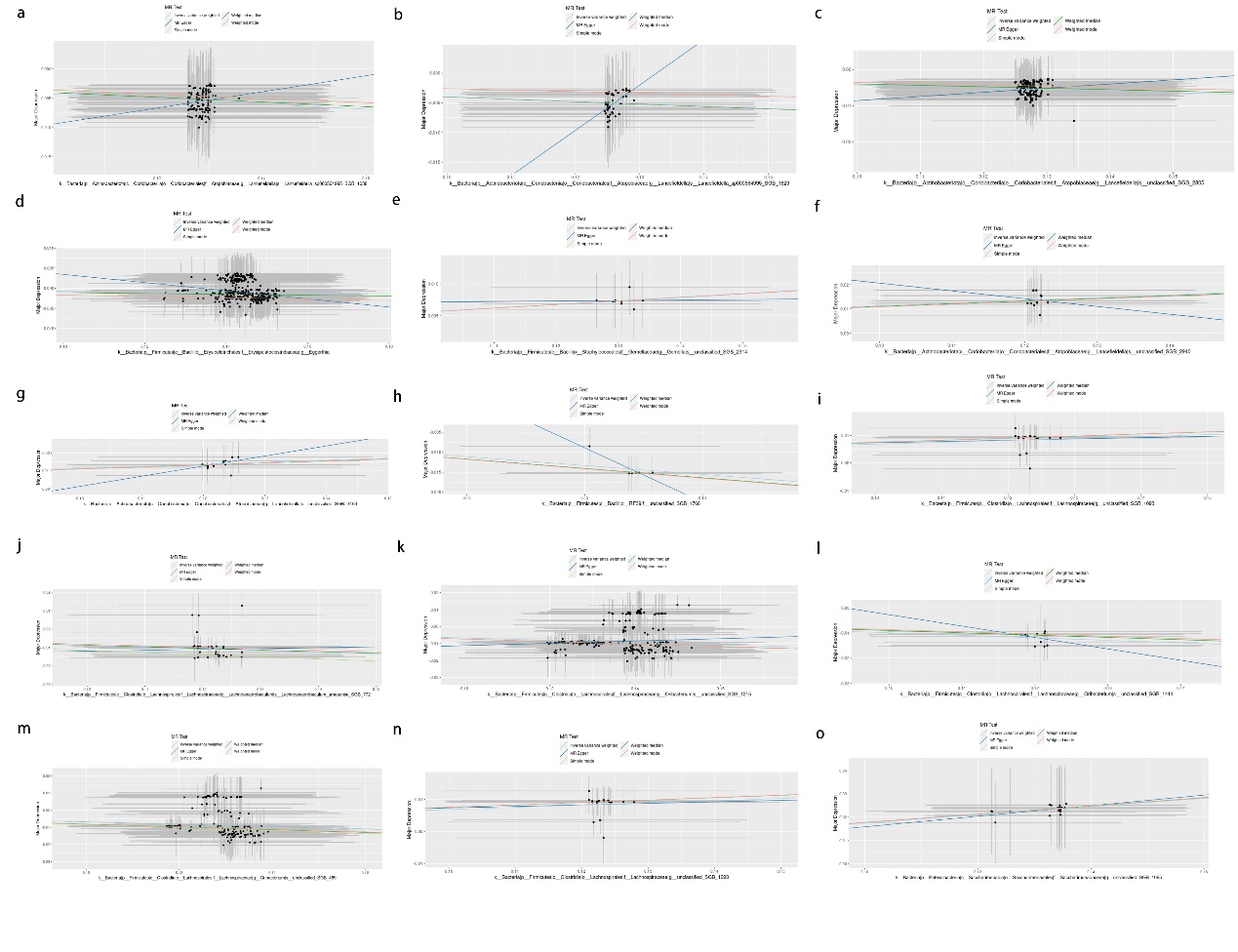


**Figure S1.** Scatter plots for MR analyses of the causal effect of oral microbiome on major depression in UK Biobank. Salivary microbiomes and dorsal tongue microbiomes represent exposure (x-axis) and major depression represents outcome (y-axis). **Figure 1a-e**: 5 salivary microbiomes. **Figure 1f-o**: 10 dorsal tongue microbiomes. For each plot we estimate the slope using Inverse variance weighted (light blue line), Weighted median (dark green line), MR Egger (navy blue line), Weighted mode (pink line), and Simple mode (light green line).


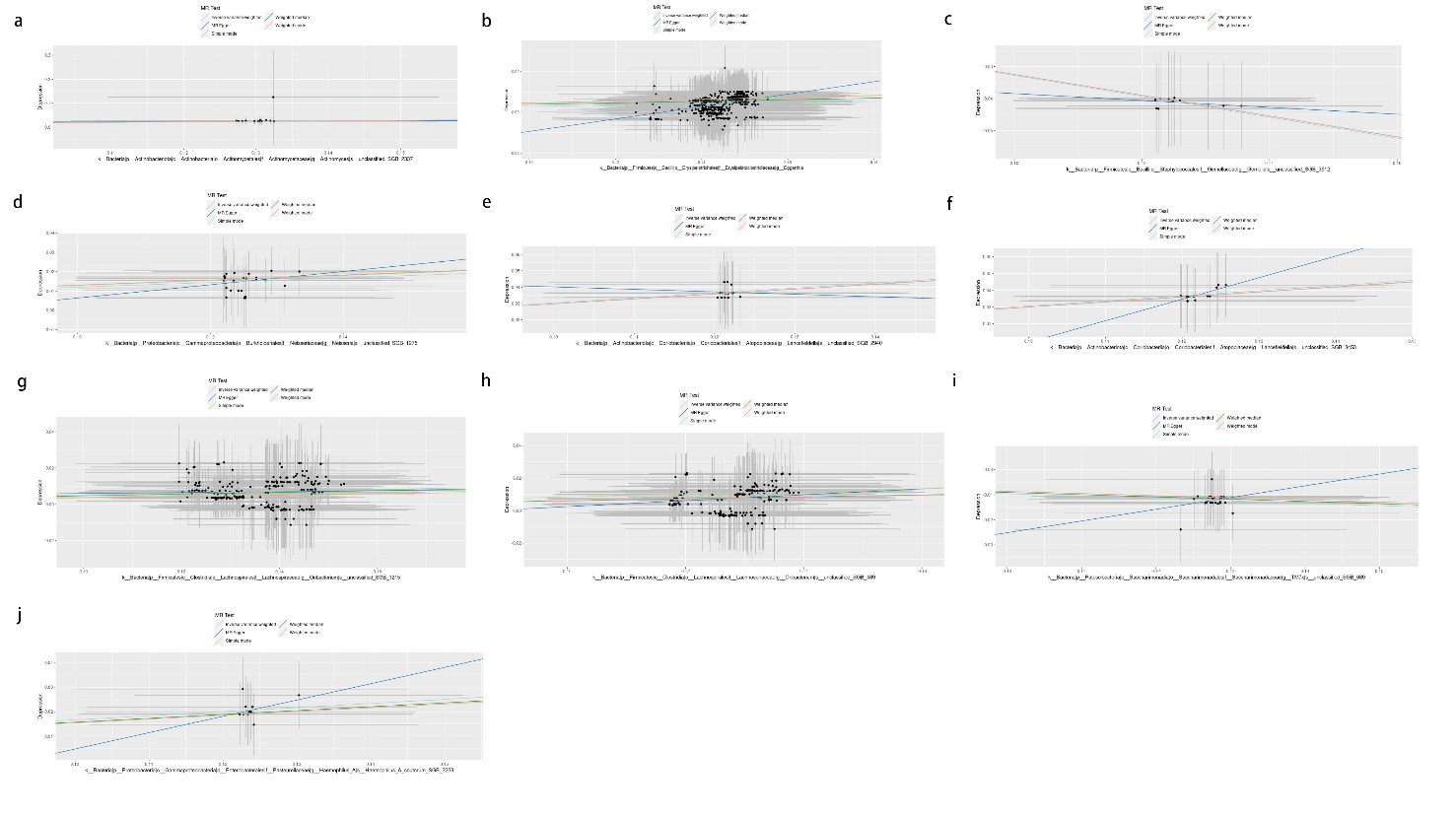


**Figure S2.** Scatter plots for MR analyses of the causal effect of oral microbiome on depression in Finngen public data. Salivary microbiomes and dorsal tongue microbiomes represent exposure (x-axis) and depression represents outcome (y-axis). **Figure S1a-d**: 4 salivary microbiomes. **Figure S1e-j**: 6 dorsal tongue microbiomes. For each plot we estimate the slope using Inverse variance weighted (light blue line), Weighted median (dark green line), MR Egger (navy blue line), Weighted mode (pink line), and Simple mode (light green line).


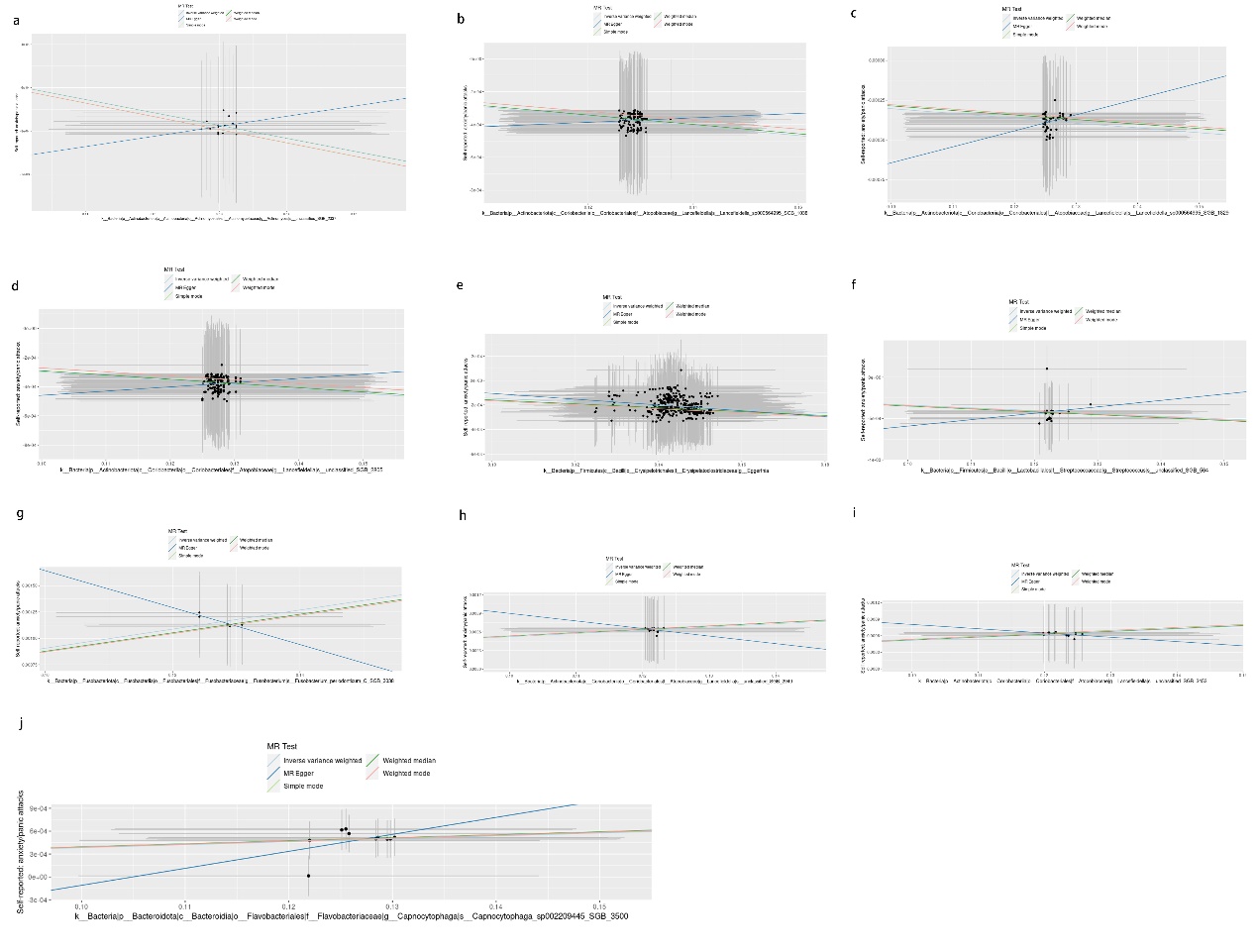


**Figure S3.** Scatter plots for MR analyses of the causal effect of oral microbiome on self-reported: anxiety/panic attacks in UK Biobank. Salivary microbiomes and dorsal tongue microbiomes represent exposure (x-axis) and self-reported: anxiety/panic attacks represent outcome (y-axis). **Figure 2a-g**: 7 salivary microbiomes. **Figure 2h-j**: 3 dorsal tongue microbiomes. For each plot we estimate the slope using Inverse variance weighted (light blue line), Weighted median (dark green line), MR Egger (navy blue line), Weighted mode (pink line), and Simple mode (light green line).


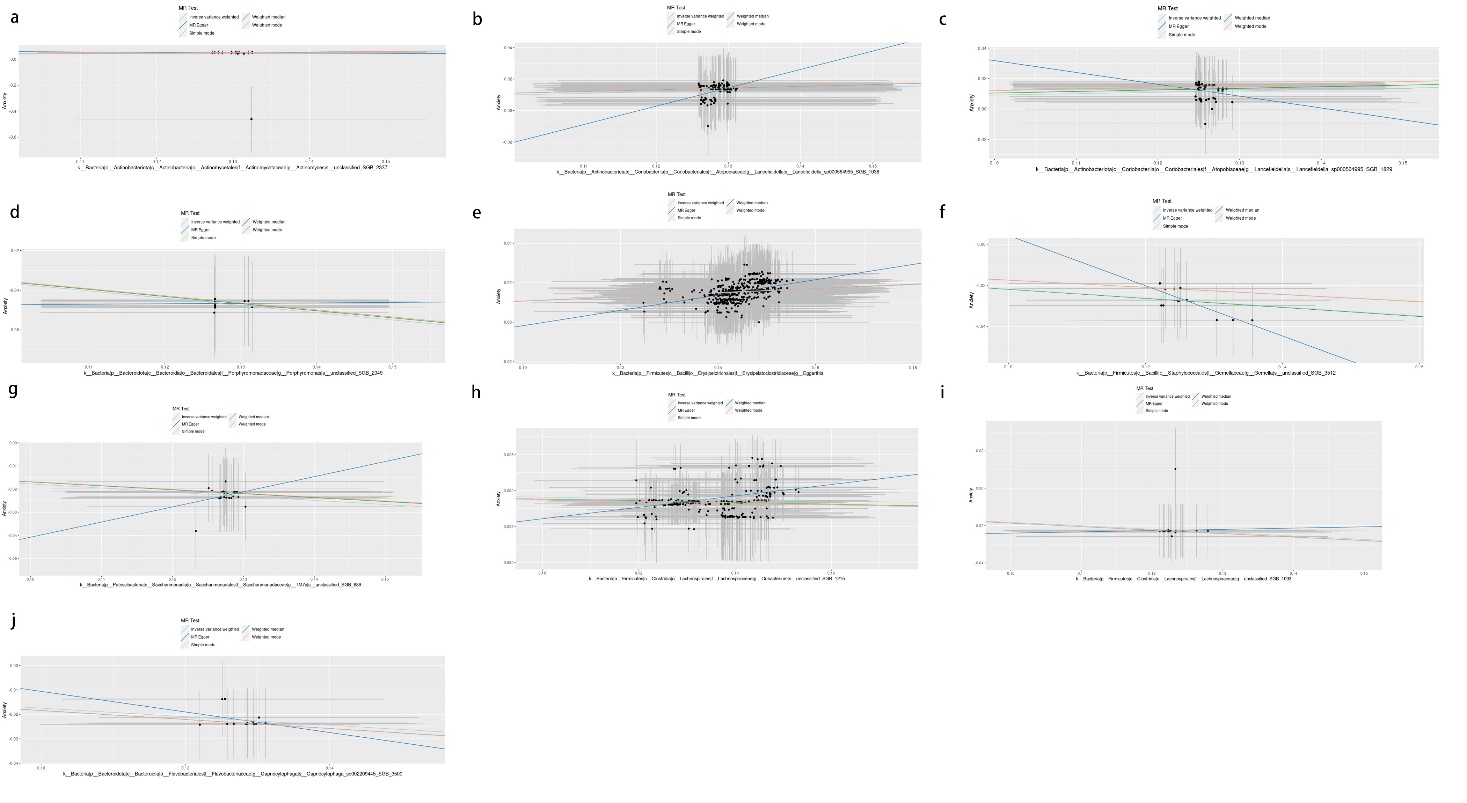


**Figure S4.** Scatter plots for MR analyses of the causal effect of oral microbiome on anxiety in Finngen public data. Salivary microbiomes and dorsal tongue microbiomes represent exposure (x-axis) and anxiety represents outcome (y-axis). **Figure S2a-f**: 6 salivary microbiomes. **Figure S2g-j**: 4 dorsal tongue microbiomes. For each plot we estimate the slope using Inverse variance weighted (light blue line), Weighted median (dark green line), MR Egger (navy blue line), Weighted mode (pink line), and Simple mode (light green line).
